# Ocular Behçet Disease—Clinical Manifestations, Treatments and Outcomes According to Age at Disease Onset

**DOI:** 10.3390/biomedicines11020624

**Published:** 2023-02-19

**Authors:** Michael Ostrovsky, Amir Rosenblatt, Salam Iriqat, Abdallah Shteiwi, Yael Sharon, Michal Kramer, Vicktoria Vishnevskia-Dai, Shaul Sar, Yosif Boulos, Oren Tomkins-Netzer, Natalie Lavee, Yael Ben-Arie-Weintrob, Hadas Pizem, Tamar Hareuveni-Blum, Marina Schneck, Raz Gepstein, Dua Masarwa, Nakhoul Nakhoul, Erez Bakshi, Shiri Shulman, Michaella Goldstein, Dan Ramon, Marina Anouk, Zohar Habot-Wilner

**Affiliations:** 1 Sackler Faculty of Medicine, Tel Aviv University, Tel Aviv 69978, Israel; 2 Division of Ophthalmology, Tel Aviv Sourasky Medical Center, Tel Aviv 6423906, Israel; 3 St. John of Jerusalem Eye Hospital Group, East Jerusalem 91198, Palestine; 4 Department of Ophthalmology, Rabin Medical Center, Petah-Tikva 4941492, Israel; 5 The Goldschleger Eye Institute, Department of Ophthalmology, Sheba Medical Center, Ramat-Gan 52620, Israel; 6 Department of Ophthalmology, Carmel Medical Center, The Ruth and Bruce Rappaport Faculty of Medicine, Technion, Israel Institute of Technology, Haifa 3436212, Israel; 7 Department of Ophthalmology, HaEmek Medical Center, The Ruth and Bruce Rappaport Faculty of Medicine, Technion, Israel Institute of Technology, Haifa 183411, Israel; 8 Department of Ophthalmology, Rambam Medical Center, The Ruth and Bruce Rappaport Faculty of Medicine, Technion, Israel Institute of Technology, Haifa 3525408, Israel; 9 Department of Ophthalmology, Galilee Medical Center, Azrieli Faculty of Medicine, Bar-Ilan University, Galilee 22100, Israel; 10 Department of Ophthalmology, Soroka University Medical Center, Ben-Gurion University of the Negev, Beer-Sheva 84101, Israel; 11 Department of Ophthalmology, Meir Medical Center, Kfar Saba 4428164, Israel; 12 Department of Ophthalmology, Barzilai Medical Center, Ashkelon, Faculty of Health Sciences, Ben-Gurion University of the Negev, Beer Sheba 7830604, Israel; 13 Department of Ophthalmology, Baruch Padeh Medical Center, Azrieli Faculty of Medicine, Bar-Ilan University, Galilee 15280, Israel; 14 Department of Ophthalmology, Assaf Harofeh Medical Center, Tzrifin 70300, Israel; 15 Ophthalmology institute, Assuta Medical Centers, Tel Aviv 6789140, Israel; 16 Faculty of Health Sciences, Ben-Gurion University of the Negev, Beer-Sheva 84105, Israel; 17 Institute of Rheumatology, Tel Aviv Sourasky Medical Center, Tel Aviv 6423906, Israel

**Keywords:** Behçet disease, ocular Behçet disease, juvenile-onset Behçet disease, adult-onset Behçet disease, late-onset Behçet disease, Behçet uveitis, uveitis

## Abstract

Behçet disease (BD) is a multisystemic disease that commonly involves the eyes. Although it affects patients in all age groups, data on ocular disease by age of onset are limited. This retrospective, multicenter study aimed to compare epidemiology, systemic and ocular manifestations, treatments and outcomes between three age groups: juvenile (<18 years), adult (18–39 years) and late (≥40 years) disease onset. The study included 175 ocular BD patients (303 eyes) from Israel and Palestine: juvenile-onset (*n* = 25, 14.3%), adult-onset (*n* = 120, 68.6%) and late-onset (*n* = 30, 17.1%). Most patients in all groups were male. Systemic manifestations were similar in all groups. Systemic co-morbidities were more common in late-onset patients. Bilateral panuveitis was the most common ocular manifestation in all patients. Non-occlusive retinal vasculitis, peripheral vessel occlusions, cataract and elevated intraocular pressure were found more commonly among juvenile-onset eyes. Anterior uveitis and macular ischemia were most common among late-onset eyes, while branch retinal vein occlusion was most common in adult and late-onset eyes. All patients were treated with corticosteroids. Methotrexate, immunomodulatory combinations and biologic treatments were more commonly used for juvenile-onset patients. All groups had a similar visual outcome. Our study showed that patients with ocular BD have varied ocular manifestations and require different treatments according to age of disease onset, but visual outcome is similar.

## 1. Introduction

Behçet disease, a chronic recurrent systemic inflammatory vascular disease, may affect blood vessels of any type and size. The disease can present with variable clinical manifestations and the most commonly involved systems are oral, ocular, cutaneous and urogenital [1,2,3,4,5,6]. Disease diagnosis is based on clinical criteria.

Patients with BD can have a genetic susceptibility for HLA-B51 and disease onset may be triggered by infectious and environmental factors [7,8,9]. BD has a unique geographic distribution, being most prevalent in countries along the ancient “silk road”, including the middle east [1].

Behçet disease typically presents in young adults, between the second and fourth decades of life. However, it can also appear during childhood or after the age of forty, with a reported prevalence of between 2% and 17% for each of these age groups [4,9,10,11,12,13]. Ocular BD commonly presents as recurrent uveitis and patients can develop various complications, leading to vision deterioration and functional impairment [14,15,16,17]. There is little literature on ocular manifestations of BD within different age groups, with no studies comparing the disease between children and adolescents, young adults and adult patients.

The aim of this study was to investigate ocular BD in a large cohort of patients, differentiating patients according to age at disease onset. The study compared the epidemiology, systemic and ocular manifestations, treatment modalities and outcomes between three age groups: juvenile-onset (<18 years), adult-onset (18–39 years) and late-onset (≥40 years).

## 2. Materials and Methods

This multicenter retrospective study included 175 patients from 13 medical centers from Israel and Palestine: 15 patients from Tel Aviv Sourasky Medical Center (0546-20), 29 patients from St. John of Jerusalem Eye hospital Group (8.2020), 25 patients from Rabin Medical Center (0765-20), 22 patients from Sheba Medical Center (7711-20), 16 patients from Carmel Medical Center (0120-20), 16 patients from HaEmek Medical center (0140-20), 14 patients from Rambam Medical Center (0397-20), 13 patients from Galilee Medical Center (0137-20), 9 patients from Soroka University Medical Center (0335-20), 9 patients from Meir Medical Center (264-20), 4 patients from Barzilai Medical Center (0040-21), 2 patients from Baruch Padeh Medical Center (0013-21) and 1 patient from Assaf Harofeh Medical Center (0353-20). It was approved by the Institutional Review Board of each participating medical center. The study included patients with ocular BD, between 2000 and 2021. All patients had at least 6 months of follow-up.

The diagnosis of BD was established according to the International Study Group for Behçet Disease (ISG) criteria [18]. In addition, all pediatric patients fulfilled the Pediatric Behçet’s Disease study (PEDBD) criteria [19]. Patients were diagnosed with ocular BD if they had ocular manifestations compatible with the disease, including: nongranulomatous anterior uveitis, diffuse vitritis, hyperemia and swelling of the optic disc, neuroretinitis, retinal vasculitis, retinal infiltrates or retinal vascular occlusions [20]. Retinal vasculitis was diagnosed clinically by the presence of perivascular sheathing and/or by vascular staining, leakage or occlusion as identified by fluorescein angiography (FA). Occlusive retinal vasculitis was diagnosed in cases with evidence of retinal capillary nonperfusion on FA. Non-occlusive retinal vasculitis cases did not have retinal capillary non-perfusion. Retinal vascular occlusions were divided into vein or artery occlusions and were specified as central, branch or peripheral occlusion by clinical appearance and FA. Inflammatory swollen disc was defined as hyperemia and blurring of optic disc margins with late leakage on FA and/or retinal nerve fiber layer thickening on optical coherence tomography (OCT). Neuroretinitis was defined as optic nerve swelling and macular oedema or macular exudates, with evidence of late leakage from the optic nerve on FA and/or macular fluid within the outer plexiform layer and/or subretinal fluid with intraretinal foci on OCT. Epiretinal membrane, macular edema and macular ischemia were diagnosed by FA and/or OCT. Cataract, optic atrophy, diffuse retinal atrophy, vitreous hemorrhage, anterior scleritis and retinal detachment were diagnosed clinically. High intraocular pressure was defined as intraocular pressure above 21 mmHg, by Goldmann applanation tonometry or iCare^®^ rebound tonometry. Uveitis was diagnosed and classified according to the Standardization of Uveitis Nomenclature (SUN) Working Group classification [21].

Epidemiologic and clinical data were obtained from medical files and included patients’ sex, age at disease onset, ethnicity (Arab, Ashkenazi Jews, Mizrahi Jews, mixed Jews), systemic comorbidities, HLA-B51 status, presence of extraocular manifestations and systemic treatments. Ocular data included laterality of eye disease, best-corrected visual acuity (BCVA), ocular manifestations, complications and ocular treatments. Treatments were retrieved at the first examination, during follow-up and at the last visit. BCVA records were collected at first and final visits, 3 months, 6 months, 1 year, 1.5 years, 2 years, 2.5 years, 3 years, 4 years, 5 years and 10 years. In addition, BCVA was stratified into 3 categories: <0.3 logMAR (better than 6/12 Snellen equivalent), 0.3–1.0 logMAR (6/12–6/60) and >1.0 logMAR (worse than 6/60), corresponding to good BCVA, moderate vision loss and severe vision loss, respectively. In Israel, children are treated by pediatric rheumatologists and ophthalmologists until the age of 18 years. Therefore, patients were divided into three groups according to their age at disease onset: juvenile-onset (age <18 years), adult-onset (18–39 years) and late-onset (age ≥40 years). All retrieved data were compared between the groups.

### Statistical Analysis

Data were recorded in Microsoft Excel 2016 and statistical analysis was performed by IBM SPSS for windows version 28.0. For analysis purposes, BCVA (Snellen) values were converted to the equivalent logarithm of the minimum angle of resolution acuity (logMAR). Two continuous variables were compared within subjects using paired t-test and between subjects using the independent sample t-test. The one-way analysis of variance (ANOVA) was used to assess differences between the means of three groups, if statistical significance was observed—a post-hoc Scheffe was used to find out comparison of which pair was significant. Fisher’s exact test or Pearson chi-square test were used to explore categorical variables between subjects. Multivariate logistic regression was used to examine the independent effect of patient demographic and clinical characteristics on moderate to severe vision loss (logMAR ≥ 0.3) at 5 years of follow-up. Variables were entered to the model if an association with a *p*-value of 0.15 or smaller was shown in the univariate analysis and no multicollinearity was found between covariates. All tests were 2-tailed and the threshold for statistical significance was defined as a *p*-value < 0.05.

## 3. Results

The study included 175 patients (303 eyes) with ocular Behçet disease: 25/175 (14.3%) juvenile-onset patients, 120/175 (68.6%) adult-onset patients and 30/175 (17.1%) late-onset patients. One-hundred twenty-five (71.4%) patients were male. Ninety-three patients (53.1%) were Arabs and 82 (46.9%) were Jewish. The Jewish patients included 2 (2.4%) Ashkenazi Jews, 20 (24.4%) mixed Jews and 60 (73.2%) Mizrahi Jews. The mean age was 28.2 ± 0.86 years (range, 4 to 60 years). The median follow-up time was 6 years (range, 0.5 to 19.8 years) and 106 patients (60.6%) had a follow-up duration of at least 5 years. There was no significant difference in follow-up duration between the groups (*p =* 0.29).

### 3.1. Demographics and Clinical Manifestations

Table 1 presents patients’ demographics and systemic manifestations according to age at disease onset. The mean age at disease onset was 12.7 ± 0.75 years in the juvenile-onset group, 26.7 ± 0.51 years in the adult-onset group and 47.1 ± 1.19 years in the late-onset group. Male sex preponderance was found in all age groups, ranging from 80.0% in the juvenile-onset group to 66.7% in the late-onset group. The late-onset group included a majority of Jewish patients (*n* = 20/30, 66.7%) as opposed to the juvenile and adult-onset groups (*n* = 12/25, 48.0% and *n* = 50/120, 41.7%, respectively, *p* < 0.05). Specifying the age groups by sub-division of Jewish ethnicity, there were fewer Mizrahi Jews in the adult-onset group (*n* = 34/120, 28.3%) as compared to the juvenile and late-onset groups (*n* = 11/25, 44.0% and *n* = 15/30, 50.0%, respectively, *p* = 0.04). Further sub-division analysis by sex and ethnicity, using independent *t*-test, showed that the mean age at diagnosis for Arab patients was 26.4 ± 1.08 years vs. 30.1 ± 1.33 years for Jewish patients (*n* = 93 and 82, respectively, *p =* 0.03). Age at diagnosis among Arab females (*n* = 26) was significantly younger than Jewish females (*n* = 24, 25.7 ± 2.12 vs. 34.0 ± 2.20 years, respectively, *p <* 0.01). HLA-B51 status rates did not differ between the groups (juvenile-onset *n* = 12/17, adult-onset *n* = 69/93, late-onset *n* = 15/23, *p =* 0.68) or between ethnicities (Jewish *n* = 42/59, 71.2%, Arab *n* = 54/74, 73.0%, *p =* 0.86).

Most patients in all groups had bilateral eye disease (*p =* 0.68). There was no difference between the groups in rate of simultaneous bilateral ocular disease (juvenile-onset *n* = 15/19, adult-onset *n* = 62/90, late-onset *n* = 14/19, *p =* 0.66), median time between unilateral presentation and second eye involvement (juvenile-onset *n* = 5, 14.1 months, adult-onset *n* = 31, 18.0 months, late-onset *n* = 6, 23.2 months, *p =* 0.59) and the likelihood of developing second eye involvement under systemic treatment (juvenile-onset *n* = 2/5, adult-onset *n* = 13/31, late-onset *n* = 4/6, *p =* 0.52).

Systemic comorbidities including hyperlipidemia, hypertension and diabetes mellitus were significantly more prevalent among patients in the late-onset group (*p* < 0.01). Oral aphthosis was found in all patients. Genital aphthosis was the second most common manifestation, followed by skin lesions. Although other systemic BD manifestations presented in different proportions between the groups, this was not found to be statistically significant.

### 3.2. Ocular Manifestations and Complications

Table 2 presents ocular manifestations and complications according to age at disease onset. Left and right eyes were similarly involved in all age groups (*p =* 0.97). Non-occlusive retinal vasculitis was the most common presentation among juvenile-onset eyes (79.5%), followed by adult-onset (61.2%) and late-onset (53.1%) eyes (*p =* 0.02).

The most common type of uveitis in all groups was panuveitis. Intermediate uveitis was found only in the adult-onset group. Late-onset eyes had significantly more anterior uveitis than in other groups (*p* < 0.01).

The juvenile-onset eyes had a significantly higher proportion of peripheral occlusions (66.7% of occlusions) compared to the adult (29.6%, *p* = 0.03) or late-onset groups (23.5%, *p* = 0.03). Branch retinal vein occlusion (BRVO) was the most common occlusion type in adult and late-onset eyes. Retinal artery occlusions were rare, and none were found in the juvenile-onset group.

The most common ocular complications in all age groups were cataract, epiretinal membrane or macular edema. Juvenile-onset eyes had significantly more cataract and high intraocular pressure (IOP) (*p* < 0.01, *p =* 0.03, respectively). Late-onset eyes had significantly more macular ischemia as compared to the other groups (*p* < 0.01). There were no cases of retinal detachment in the juvenile-onset eyes. Anterior scleritis was not found in late-onset eyes.

### 3.3. Visual Acuity

**Figure 1:**
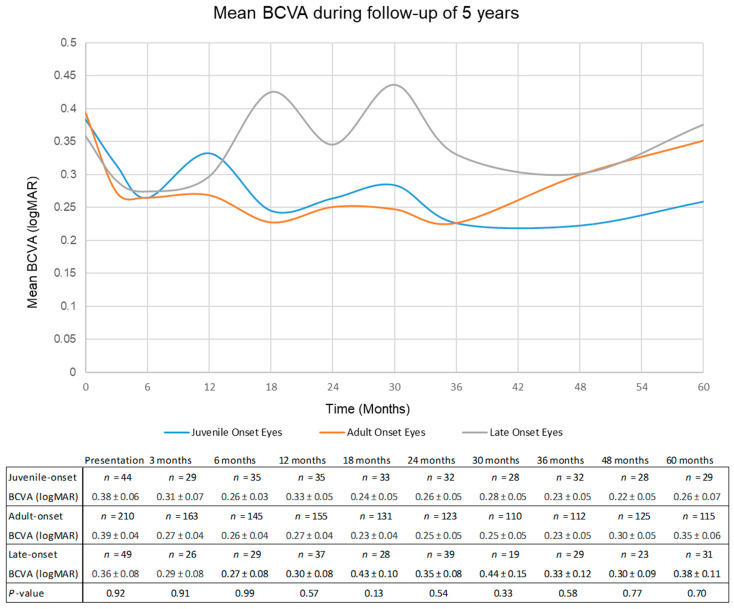
Mean best-corrected visual acuity from baseline to 60 months. ANOVA test was performed to compare the best-corrected visual acuity between all groups at each time point. There was no statistically significant difference between the groups at all time points.

Figure 1 illustrates the mean logMAR BCVA during follow-up among the different age groups. Mean BCVA for the whole cohort was 0.39 logMAR at presentation and 0.34 logMAR at 60 months. Comparing between the age groups using ANOVA, there was no difference in BCVA at presentation between juvenile (*n* = 44), adult (*n* = 210) and late-onset (*n* = 49) eyes (0.38 ± 0.06, 0.39 ± 0.04 and 0.36 ± 0.08 logMAR, respectively, *p =* 0.92). At 60 months follow-up, juvenile-onset eyes had better mean BCVA (*n* = 29, 0.26 ± 0.07 logMAR) compared to the other groups (*n* = 115, 0.35 ± 0.06 and *n* = 31, 0.37 ± 0.10 logMAR), but this was not statistically significant (*p =* 0.70).

One-hundred and six patients (including 33 juvenile-onset eyes, 125 adult-onset eyes and 34 late-onset eyes) had a follow-up time of at least 60 months. At presentation, mean BCVA was better than 20/40 in 17 (51.5%) juvenile-onset eyes, 77 (61.6%) adult-onset eyes and 25 (73.5%) late-onset eyes (*p =* 0.18); between 20/40 and 20/200 in 14 (42.4%) juvenile-onset eyes, 36 (28.8%) adult-onset eyes and 8 (23.5%) late-onset eyes (*p =* 0.20) and was worse than 20/200 in 2 (6.1%) juvenile-onset eyes, 12 (9.6%) adult-onset eyes and 1 (2.9%) late-onset eye (*p =* 0.40). At 60 months, mean BCVA was better than 20/40 in 21 (63.6%) juvenile-onset eyes, 86 (68.8%) adult-onset eyes and 23 (67.6%) late-onset eyes (*p =* 0.85). Mean BCVA was between 20/40 and 20/200 in 10 (30.3%) juvenile-onset eyes, 30 (24.0%) adult-onset eyes and 8 (23.5%) late-onset eyes (*p =* 0.74). Mean BCVA was worse than 20/200 in 2 (6.1%) juvenile-onset eyes, 9 (7.2%) adult-onset eyes and 3 (8.8%) late-onset eyes (*p =* 0.91). A univariate analysis was performed to examine demographics and clinical characteristics associated with moderate to severe vision loss (logMAR ≥0.3) at 60 months follow-up. Unilateral disease (*p* < 0.01), Arab ethnicity (*p* = 0.05), the presence of retinal vascular occlusions (*p* < 0.01), neovascularization elsewhere (NVE) (*p* < 0.01), neovascularization of the disc (NVD) (*p* = 0.05), VH (*p* < 0.01), foveal ischemia (*p* < 0.01), diffuse retinal atrophy (*p* = 0.04) and moderate to severe visual loss on presentation (*p* = 0.01) were found to be significantly more common in patients with vision loss.

These variables, as well as cataract (*p* = 0.07), were entered into a multivariate logistic regression analysis model. Significant independent factors were the presence of retinal vascular occlusions (*p* = 0.05, Odds ratio = 3.5) and moderate to severe visual loss on presentation (*p* < 0.01, Odds ratio = 25.0). In this model, age of disease onset did not show an independent association with vision loss.

### 3.4. Systemic and Surgical Treatments

Table 3 demonstrates systemic treatments during follow-up and at the final visit for patients with a follow-up >60 months. All patients were treated with systemic corticosteroids; all patients were given oral prednisone, with intravenous methylprednisolone administered to a third of the juvenile-onset patients and to a quarter of the adult and late-onset patients (*p =* 0.53).

Immunomodulatory (IMN) treatments included Cyclosporin, Methotrexate, Azathioprine and Mycophenolate Mofetil. All juvenile-onset patients, 85.0% of adult-onset patients and 80.0% of late-onset patients received IMN treatments (*p =* 0.05). Juvenile-onset patients were more likely to receive methotrexate (72.0%) than adult or late-onset patients (40.0% and 36.7%, respectively, *p* < 0.01). No patient in the juvenile-onset group was treated with Mycophenolate Mofetil. IMN treatment types were similar among the adult and late-onset patients. Combined IMN treatment was significantly more common among patients in the juvenile-onset group (*p =* 0.01). There was no significant difference in IMN treatment duration between the groups (*p =* 0.29).

Most of the juvenile-onset patients were treated with biologic treatments (68.0%) as compared to 50.8% of the adult-onset patients and 36.7% of late-onset patients (*p =* 0.04). Adalimumab was the most commonly used biologic treatment in all age groups, followed by Infliximab. Other biologic treatments included Tocilizumab, Rituximab and Interferon α and were rarely used. There was no significant difference between the groups regarding biologic treatment switch (*p =* 0.96). Biologic treatment duration was longest among juvenile-onset patients (311.0 weeks), followed by patients in the adult-onset group (169.9 weeks) and was shortest for the late-onset patients (158.9 weeks) (*p =* 0.02). Furthermore, post hoc analysis showed significant differences in treatment duration between the juvenile and adult-onset groups (*p* < 0.01) and between the juvenile and late-onset groups (*p* < 0.01). There was no difference between the adult and late-onset groups (*p* = 0.86).

Treatments at last visit were analyzed for 106 patients with a follow-up of > 60 months. Significantly fewer patients in the late-onset group were treated at final visit (*p =* 0.02). Overall, 72 (67.9%) patients were corticosteroid-free. Patients in the adult-onset group were more likely to use prednisone (41.8%, *p* = 0.02), with 28.4% receiving a dosage above 7.5 mg per day (median dose 20 mg prednisone). Furthermore, adult-onset patients were also more likely to use IMN treatments (53.7%), significantly more compared to late-onset patients (28.6%, *p =* 0.04). Methotrexate was most commonly used by juvenile-onset patients, while azathioprine was most commonly used by adult and late-onset patients. Only juvenile-onset patients received IMN combination (azathioprine and cyclosporine). Biologic treatment was more commonly used by juvenile-onset patients compared to the other groups (*p =* 0.03).

Fifty-eight of 303 eyes (19.1%) underwent ocular surgeries: 8 (18.2%) juvenile-onset eyes, 33 (15.7%) adult-onset eyes and 17 (34.7%) late-onset eyes (*p =* 0.01). The most common ocular surgery was cataract extraction with intraocular lens implantation (*n* = 49/58, 84.5%). There were significantly more operations for late-onset eyes (*n* = 15/49, 30.6%) than juvenile or adult-onset eyes (*n* = 8/44, 18.2% and *n* = 26/210, 12.4%, respectively, *p* < 0.01). Intravitreal avastin injections were used significantly more in eyes in the late-onset group (*n* = 10/30, 20.4%) compared to juvenile or adult-onset eyes (*n* = 3/25, 6.8% and *n* = 9/120, 4.3%, respectively, *p* < 0.01).

## 4. Discussion

This study analyzed a large group of patients with ocular Behçet disease according to their age at disease onset. To the best of our knowledge, this is the first study comparing ocular BD patients in all age groups, highlighting disease findings and outcomes in different age populations. Epidemiological and clinical characteristics, treatment modalities and outcomes were compared between three groups: juvenile-onset (age < 18 years), adult-onset (18–39 years) and late-onset (age ≥ 40 years).

Our study included 175 patients (303 eyes) with an equal distribution between juvenile and late-onset patients (each group included ~15% of patients) and the largest group was adult-onset patients. Up to now, only a few studies have reported on ocular BD according to a specific age group: 4 studies on juvenile-onset patients [10,22,23,24] and 4 studies on late-onset patients [13,25,26,27]. None of these studies compared between all different age groups, hence data on differences between all age groups are sparse.

In our study, most patients in all groups were male, but the male to female ratio decreased as age at disease onset increased. This finding is in concordance with previous reports that also showed that ocular BD is more common among male patients, who also typically have a younger age of disease onset [1,15,17]. Jewish patients were significantly more prevalent in the late-onset group and Arab female patients presented at the youngest age. To note, HLA-B51 was found similarly in all age groups and ethnicities and could not be connected to this finding.

There were several publications demonstrating correlations between systemic BD and systemic comorbidities including hyperlipidemia, hypertension and diabetes. A Turkish study [28] showed BD to be an independent risk factor for metabolic syndrome (central obesity, elevated triglycerides, reduced high-density lipoproteins, impaired fasting blood glucose and hypertension), with BD patients having a 2.67-fold risk when compared to healthy controls. Furthermore, this study showed that patient’s age and age at disease onset significantly correlated with increased risk of metabolic syndrome. However, in this study less than a third of the patients had ocular involvement, which was not found to be correlated to metabolic syndrome. Another Turkish study [29] compared BD patients to healthy controls and found fasting blood sugar, insulin levels and diastolic blood pressure to be significantly higher in BD patients. Our study is the first to analyze the relation between ocular BD and systemic comorbidities according to age of disease onset. We found hyperlipidemia, hypertension and diabetes to be more common among late-onset ocular BD patients. This finding may be explained by the well-established effect of aging on cardiovascular disease and highlights the significance of close monitoring for comorbidities and encouraging patients to adopt a healthy lifestyle.

Oral aphthosis was found in all patients. Although ratios of systemic manifestations varied between the age groups, overall, no significant difference was found. Similarly, Kitaichi et al. [10] did not find different rates of cutaneous disease between ocular BD patients younger and older than 16 years.

Most patients had bilateral eye disease, as was previously reported in patients with ocular BD [10,13,15,17,22,23,27]. Panuveitis was the most common type of uveitis in all groups, involving approximately half of the eyes among juvenile and adult-onset patients and about a third of late-onset eyes. Anterior uveitis was significantly more common among late-onset eyes. Intermediate uveitis was found as a sole uveitis manifestation only in adult-onset eyes. Kitaichi et al. [10], Tugal-Tuktun et al. [23] and Kramer et al. [24] reported on ocular BD among juvenile-onset patients from different continents and also found bilateral panuveitis to be the most common type of uveitis. Sungur et al. [13] and Hazirolan et al. [25] from Turkey reported on ocular BD among late-onset BD patients and found anterior uveitis to be the most common uveitis type. However, similar to our findings, a study from Tunisia [26] on ocular late-onset BD patients found that panuveitis was the most common uveitis type (43%), followed by anterior uveitis (33%).

Non-occlusive retinal vasculitis, although common in all age groups, was more common in the juvenile-onset group and its prevalence declined as age of onset increased. Other studies did not specify retinal vasculitis as occlusive or non-occlusive and therefore could not be compared to our results. Retinal vascular occlusions were found in all age groups and their prevalence increased with age. The most common occlusion type in adult and late-onset eyes was BRVO, while peripheral vessels occlusion was the most common type in juvenile-onset eyes. Retinal artery occlusions were rare and were not found in the juvenile-onset group. Only one study on juvenile-onset eyes [23] reported a single case of BRVO and one study on late-onset eyes [13] reported two BRVO cases.

The most common ocular complications in all age groups were cataract, epiretinal membrane or macular edema. We found that juvenile-onset eyes were more prone to develop cataract (61%) and elevated IOP (25%), while late-onset eyes had a greater risk of macular ischemia, which might correlate to their slightly higher rate of retinal vascular occlusions. Other studies reported an incidence of cataract ranging between 27% and 49% in juvenile-onset eyes [23,24].

Mean best corrected visual acuity at presentation was similar among all age groups. Looking on long-term visual outcome at 5 years follow-up, juvenile-onset eyes had mildly better mean BCVA compared to the other groups. This could be explained by unique findings in the juvenile-onset group: a higher proportion of peripheral vascular occlusions, absence of arterial occlusions, lower incidence of macular ischemia and absence of retinal detachment. Age of disease onset was not found to have an independent effect on visual outcome by multivariate analysis.

All patients were treated with oral corticosteroids. More juvenile-onset patients received intravenous methylprednisolone and combined IMN treatments compared to the other groups. This difference may be related to a more severe ocular disease among juvenile-onset patients and the use of “aggressive” treatments to achieve disease quiescence. Regarding the use of IMN drugs, methotrexate was most commonly used by juvenile-onset patients while azathioprine was the most common drug used by other groups. This reflects the common treatment practice for different age groups in Israel. Overall, biologic treatments were more commonly used in our study compared to previous publications [10,13,23,25,26,27]. Biologic treatments, particularly anti-TNFs, are becoming more popular for treating ocular BD. An expert panel recommended considering the use of anti-TNF agents [30], particularly infliximab and adalimumab, as first-line immunomodulatory agents for the treatment of ocular BD. Biologic treatments were used in our cohort in two-thirds of juvenile-onset patients, about a half of the adult-onset and a third of late-onset patients. Adalimumab was the most commonly used biologic treatment in all age groups. Biologic treatment duration was longest among juvenile-onset patients (311 weeks), followed by patients in the adult-onset group (170 weeks) and was shortest for late-onset patients (159 weeks). As all groups had a similar length of follow-up, this fact may reflect the early use of anti-TNF treatments among juvenile-onset patients and could be explained by a more severe ocular disease in this group. Intravitreal avastin injections were significantly more commonly used in eyes in the late-onset group, in concordance with their higher rate of retinal vein occlusions and vitreous hemorrhage.

We examined long-term treatment modalities at the last visit of patients with a follow-up of more than 5 years. We found that nearly a third of our patients did not receive any treatment and two-thirds were corticosteroid-free. Significantly fewer patients in the late-onset group received treatment. Adult-onset patients were significantly more likely to receive IMN treatment compared to late-onset patients. Juvenile-onset patients used more biologic treatments, particularly infliximab. Overall, these results highlight good long-term disease control and ocular outcome with most patients not using corticosteroids.

About a fifth of the eyes in our cohort underwent ocular surgeries, with late-onset eyes over twice as likely to undergo surgery than eyes in the other groups. The most common ocular surgery was cataract extraction with intraocular lens implantation. This is to be expected due to cataract progression over the years with the presence of significant cataract among older patients.

Our study’s main limitation was its retrospective design. The ISG criteria for diagnosis of BD, as well as the PEDBD criteria for juvenile-onset patients, were used in patient selection to reduce selection bias. Our patients had a follow-up of at least 6 months in order to avoid any possible bias stemming from a short follow-up. Indeed, nearly two-thirds of our patients had a follow-up time of over 5 years.

## 5. Conclusions

The present study is the first to analyze patients with ocular BD in three different age groups by an evaluation of epidemiology, clinical characteristics, treatment modalities and outcomes. In our cohort, the adult-onset group constituted the majority (~2/3) of patients, while the juvenile and late-onset groups had a similar prevalence. Systemic manifestations and visual acuity outcome were similar among all groups, but ocular manifestations and complications differed. Diverse treatments were used in different age groups. Ophthalmologists should be aware that ocular BD may differ between age groups and additional studies are warranted to further assess such differences.

## Figures and Tables

**Table 1:** Demographics and clinical manifestations.

	Juvenile Onset (*n* = 25)	Adult Onset (*n* = 120)	Late Onset (*n* = 30)	*p*-value	Total
Age at disease onset - years (mean ± SEM)	12.7 ± 0.75	26.7 ± 0.51	47.1 ± 1.19	<0.01 *	28.2 ± 0.86
Sex - male, *n* (%)	20 (80.0%)	85 (70.8%)	20 (66.7%)	0.53 ˆ	125 (71.4%)
Ethnicity - Jewish, *n* (%)	12 (48.0%)	50 (41.7%)	20 (66.7%)	<0.05 ˆ	82 (46.9%)
HLA-B51 - positive, *n* (%)	12 (70.6%)	69 (74.2%)	15 (65.2%)	0.68 ˆ	96 (72.2%)
Systemic disease before ocular presentation, *n* (%)	8 (32.0%)	49 (42.2%)	8 (26.7%)	0.23 ˆ	65 (37.1%)
Systemic disease duration before ocular presentation -months (mean ± SEM)	23.2 ± 8.03	49.3 ± 8.92	64.7 ± 37.09	0.47 *	49.8 ± 7.99
Bilateral eye disease, *n* (%)	19 (76.0%)	90 (75.0%)	19 (63.3%)	0.68 ˆ	128 (73.1%)
Follow-up duration - months (mean ± SEM)	102.9 ± 12.16	81.3± 6.08	87.2 ± 8.31	0.29 *	85.4 ± 4.75
Systemic comorbidities, *n* (%)					
Hyperlipidemia	3 (12.0%)	20 (16.7%)	18 (60.0%)	<0.01 ˆ	41 (23.4%)
Hypertension	2 (8.0%)	12 (10%)	17 (56.7%)	<0.01 ˆ	31 (17.7%)
Diabetes mellitus	1 (4.0%)	10 (8.3%)	13 (43.3%)	<0.01 ˆ	24 (13.7%)
Systemic manifestations, *n* (%)					
Oral aphthosis	25 (100.0%)	120 (100.0%)	30 (100.0%)	1.00 ˆ	175 (100.0%)
Genital aphthosis	13 (52.0%)	66 (55.0%)	13 (43.3%)	0.52 ˆ	92 (52.6%)
Skin lesions	12 (48.0%)	41 (34.2%)	11 (36.7%)	0.43 ˆ	64 (36.6%)
Arthritis	3 (10.7%)	24 (24.8%)	10 (33.3%)	0.13 ˆ	37 (25.2%)
Neurological manifestations	2 (8.0%)	13 (10.8%)	5 (16.7%)	0.56 ˆ	20 (11.4%)
Vascular manifestations	2 (8.0%)	16 (13.3%)	2 (6.7%)	0.50 ˆ	20 (11.4%)
Epididymitis	1 (3.6%)	9 (8.0%)	3 (10.0%)	0.70 ˆ	13 (8.8%)
Gastrointestinal lesions	0 (0.0%)	1 (0.8%)	1 (3.3%)	0.43 ˆ	2 (1.1%)

* Continuous variables were compared between the groups using the one-way analysis of variance (ANOVA). ˆ Categorical variables were compared between the groups using Fisher’s exact test or Pearson chi-square test. SEM = Standard error of the mean, HLA = Human leukocyte antigen.

**Table 2:** Ocular manifestations and complications.

	Juvenile Onset (*n* = 44)	Adult Onset (*n* = 210)	Late Onset (*n* = 49)	*p*-Value	Total
Left eye involvement, *n* (%)	22 (50.0%)	109 (51.9%)	25 (51.0%)	0.97	156 (51.5%)
Non-occlusive retinal vasculitis, *n* (%)	35 (79.5%)	128 (61.2%)	26 (53.1%)	0.02	189 (62.6%)
Neuroretinitis and inflammatory swollen disc, *n* (%)	17 (38.6%)	57 (27.1%)	10 (22.4%)	0.14	84 (27.7%)
Type of uveitis at presentation, *n* (%)					
Anterior	6 (13.6%)	20 (9.5%)	15 (30.6%)	<0.01	41 (13.5%)
Intermediate	0 (0.0%)	15 (7.1%)	0 (0.0%)	0.03	15 (5.0%)
Posterior	3 (6.8%)	38 (17.1%)	5 (10.2%)	0.09	46 (15.2%)
Panuveitis	26 (59.1%)	107 (51.0%)	18 (36.7%)	0.08	151 (49.8%)
ANT + INT	1 (2.3%)	16 (7.6%)	2 (4.1%)	0.33	19 (6.3%)
ANT + POST	0 (0.0%)	4 (1.9%)	5 (10.2%)	0.01	9 (3.0%)
INT + POST	8 (18.2%)	10 (4.8%)	4 (8.2%)	<0.01	22 (7.3%)
Retinal vascular occlusions, *n* (%)					
Any type of occlusion	9 (20.5%)	54 (25.7%)	17 (34.7%)	0.27	80 (26.4%)
CRVO	0 (0.0%)	7 (3.3%)	4 (8.2%)	0.09	11 (3.6%)
BRVO	3 (6.8%)	29 (13.8%)	9 (18.4%)	0.26	41 (13.5%)
Peripheral vessels	6 (13.6%)	16 (7.6%)	4 (8.2%)	0.43	26 (8.6%)
CRAO	0 (0.0%)	0 (0.0%)	1 (2.0%)	0.30	1 (0.3%)
BRAO	0 (0.0%)	5 (2.4%)	0 (0.0%)	0.64	5 (1.7%)
Complications, *n* (%)					
Cataract	27 (61.4%)	70 (33.3%)	22 (44.9%)	<0.01	119 (39.3%)
Epiretinal membrane	17 (38.6%)	68 (32.4%)	14 (28.6%)	0.58	99 (32.7%)
Macular edema	16 (36.4%)	60 (28.6%)	19 (38.8%)	0.28	95 (31.4%)
Macular ischemia	3 (6.8%)	24 (11.4%)	13 (26.5%)	<0.01	40 (13.2%)
High IOP	11 (25.0%)	22 (10.5%)	6 (12.2%)	0.03	39 (12.9%)
Diffuse retinal atrophy	5 (11.4%)	14 (6.7%)	6 (12.2%)	0.32	25 (8.3%)
Optic atrophy	6 (13.6%)	13 (6.2%)	3 (6.1%)	0.21	22 (7.3%)
Vitreous hemorrhage	3 (6.8%)	11 (5.2%)	7 (14.3%)	0.08	21 (6.9%)
Anterior scleritis	1 (2.3%)	9 (4.3%)	0 (0.0%)	0.29	10 (3.3%)
Retinal detachment	0 (0.0%)	5 (2.4%)	3 (6.1%)	0.17	8 (2.6%)

Categorical variables were compared between the groups using Fisher’s exact test or Pearson chi-square test. ANT = Anterior, INT = Intermediate, POST = Posterior, CRVO = Central retinal vein occlusion, BRVO = Branch retinal vein occlusion, CRAO = Central retinal artery occlusion, BRAO = Branch retinal artery occlusion, IOP = Intra-ocular pressure.

**Table 3:** Treatments.

	Juvenile Onset (*n* = 25)	Adult Onset (*n* = 120)	Late Onset (*n* = 30)	*p*-Value	Total
Prednisone, *n* (%)	25 (100.0%)	120 (100.0%)	30 (100.0%)	1.00 ˆ	175 (100.0%)
Intravenous pulse methylprednisolone, *n* (%)	9 (36.0%)	30 (25.0%)	8 (26.7%)	0.53 ˆ	47 (26.9%)
IMN treatment, *n* (%)	25 (100.0%)	102 (85.0%)	24 (80.0%)	<0.05 ˆ	151 (86.3%)
Cyclosporine	11 (44.0%)	32 (26.7%)	6 (20.0%)	0.12 ˆ	49 (28.0%)
Methotrexate	18 (72.0%)	48 (40.0%)	11 (36.7%)	<0.01 ˆ	77 (44.0%)
Azathioprine	15 (60.0%)	73 (60.8%)	18 (60.0%)	0.99 ˆ	106 (60.6%)
Mycophenolate mofetil	0 (0.0%)	6 (5.0%)	1 (3.3%)	0.84 ˆ	7 (4.0%)
IMN combination, *n* (%)	9 (36.0%)	14 (11.7%)	5 (16.7%)	0.01 ˆ	28 (16.0%)
IMN switch, *n* (%)	11 (44.0%)	33 (27.5%)	6 (20.0%)	0.13 ˆ	50 (28.6%)
IMN treatment duration - weeks (mean ± SEM)	239.9 ± 52.2	254.4 ± 27.2	187.9 ± 40.5	0.29 *	243.1 ± 23.1
Biologic treatment, *n* (%)	17 (68.0%)	61 (50.8%)	11 (36.7%)	0.04 ˆ	89 (50.9%)
Infliximab	8 (32.0%)	17 (14.2%)	4 (13.3%)	0.08 ˆ	29 (16.6%)
Adalimumab	11 (44.0%)	52 (43.3%)	8 (26.7%)	0.23 ˆ	71 (40.6%)
Other biologic treatments(Tocilizumab, rituximab, interferon α)	1 (4.0%)	1 (0.8%)	1 (3.3%)	0.41 ˆ	3 (1.7%)
Biologic treatment switch, *n* (%)	2 (8.0%)	10 (8.3%)	2 (6.7%)	0.96 ˆ	14 (8.0%)
Biologic treatment duration - weeks (mean ± SEM)	311.0 ± 54.9	169.9 ± 23.6	158.9 ± 46.5	0.02 *	195.8 ± 20.9
Intravitreal anti-VEGF (avastin) injection, *n* (%)	3 (6.8%)	9 (4.3%)	10 (20.4%)	<0.01 ˆ	22 (7.3%)
Treatment at final visit(patients with follow-up >5 years)	Juvenile Onset (*n* = 18)	Adult Onset (*n* = 67)	Late Onset (*n* = 21)	*p*-value	Total
Any Treatment, *n* (%)	14 (77.8%)	49 (73.1%)	9 (42.9%)	0.02 ˆ	72 (67.9%)
Prednisone, *n* (%)	3 (16.7%)	28 (41.8%)	3 (14.3%)	0.02 ˆ	34 (32.1%)
Prednisone > 7.5mg/day, *n* (%)	1 (5.6%)	19 (28.4%)	2 (9.5%)	0.04 ˆ	22 (20.8%)
IMN treatment, *n* (%)	6 (33.3%)	36 (53.7%)	6 (28.6%)	0.09 ˆ	48 (45.3%)
Cyclosporine	1 (5.6%)	1 (1.5%)	1 (4.8%)	0.55 ˆ	3 (2.8%)
Methotrexate	3 (16.7%)	10 (14.9%)	0 (0.0%)	0.15 ˆ	13 (12.3%)
Azathioprine	1 (5.6%)	15 (22.4%)	4 (19.0%)	0.27 ˆ	20 (18.9%)
Mycophenolate mofetil	0 (0.0%)	3 (4.5%)	1 (4.8%)	0.99 ˆ	4 (3.8%)
IMN combination, *n* (%)					
Azathioprine + cyclosporin	1 (5.6%)	0 (0.0%)	0 (0.0%)	0.17 ˆ	1 (0.9%)
Biologic treatment, *n* (%)	10 (55.6%)	25 (37.3%)	4 (19.0%)	0.03 ˆ	39 (36.8%)
Infliximab	6 (33.3%)	8 (11.9%)	2 (9.5%)	0.06 ˆ	16 (15.1%)
Adalimumab	4 (22.2%)	17 (25.4%)	1 (4.8%)	0.13 ˆ	22 (20.8%)
Actemra	0 (0.0%)	0 (0.0%)	1 (4.8%)	0.36 ˆ	1 (0.9%)
IMN + biologic treatment, *n* (%)	2 (11.1%)	15 (22.4%)	2 (9.5%)	0.29 ˆ	19 (17.9%)
IMN + prednisone treatment, *n* (%)	2 (11.1%)	20 (29.9%)	3 (14.3%)	0.13 ˆ	25 (23.6%)
Prednisone + biologic treatment, *n* (%)	1 (5.6%)	10 (14.9%)	1 (4.8%)	0.31 ˆ	12 (11.3%)

* Continuous variables were compared between the groups using the one-way analysis of variance (ANOVA). ˆ Categorical variables were compared between the groups using Fisher’s exact test or Pearson chi-square test. IMN = Immunomodulatory, VEGF = Vascular endothelial growth factor.

## Data Availability

No additional data are available.
